# Scalable Predictions for Spatial Probit Linear Mixed Models Using Nearest Neighbor Gaussian Processes

**DOI:** 10.6339/22-jds1073

**Published:** 2022-11-03

**Authors:** Arkajyoti Saha, Abhirup Datta, Sudipto Banerjee

**Affiliations:** 1 Department of Statistics, University of Washington, Seattle, WA, USA; 2 Department of Biostatistics, Johns Hopkins University, Baltimore, MD, USA; 3 UCLA Department of Biostatistics, 650 Charles E. Young Drive South, University of California Los Angeles, CA 90095-1772, USA

**Keywords:** binary data, generalized linear mixed models, spatial, Gaussian processes

## Abstract

Spatial probit generalized linear mixed models (spGLMM) with a linear fixed effect and a spatial random effect, endowed with a Gaussian Process prior, are widely used for analysis of binary spatial data. However, the canonical Bayesian implementation of this hierarchical mixed model can involve protracted Markov Chain Monte Carlo sampling. Alternate approaches have been proposed that circumvent this by directly representing the marginal likelihood from spGLMM in terms of multivariate normal cummulative distribution functions (cdf). We present a direct and fast rendition of this latter approach for predictions from a spatial probit linear mixed model. We show that the covariance matrix of the cdf characterizing the marginal cdf of binary spatial data from spGLMM is amenable to approximation using Nearest Neighbor Gaussian Processes (NNGP). This facilitates a scalable prediction algorithm for spGLMM using NNGP that only involves sparse or small matrix computations and can be deployed in an embarrassingly parallel manner. We demonstrate the accuracy and scalability of the algorithm via numerous simulation experiments and an analysis of species presence-absence data.

## 1 Introduction

Spatial analysis of non-Gaussian outcomes is widely prevalent in diverse fields within the natural and environmental sciences (De Oliveira et al., 1997; Diggle et al., 1998; Zhang et al., 2022). In several instances we measure variables that are not naturally modeled as Gaussian and may not even be continuous. For example, the outcome of interest might be a spatially referenced binary variable measuring whether the rainfall at a given spatial location was above a specified threshold or not. In ecological and forestry applications, data are collected at spatial locations indicating whether a particular species is present or absent at that location. First proposed in Heagerty and Lele (1998), De Oliveira (2000), there is, by now, a considerable literature on the modeling and analysis of spatial non-Gaussian, and binary in particular, data for which a comprehensive review is beyond the scope of this article. We will focus on settings where the outcome can reasonably be treated as point-referenced and modeled using a stochastic process embedded within a hierarchical model. Spatial referencing for non-Gaussian data may sometimes be aggregated into rates or counts over larger regions, but high-resolution spatial data compiled over areas that are sufficiently small are often analyzed using process-based methods for point-referenced data (see, e.g. Diggle et al., 1998).

Following Diggle et al. (1998), we can introduce spatially dependent stochastic processes for non-Gaussian data as follows. Let Y(s) be the outcome of interest at location s, where the probability law for Y(s) cannot be reasonably assumed to be Gaussian. Therefore, we endow Y(s) with a probability law corresponding to a member of the exponential family of densities,

(1) f(Y(s)∣β,w(s),γ)=h(y(s),γ)×exp{γ[y(s)η(s)−ψ(η(s))]},

where γ is a dispersion parameter, $g(\eta (s))=x(s{)}^{\prime }\beta +w(s)$ for specified real-valued link function g(⋅), w(s) is a spatial random effect at an arbitrary location s assumed to be realized from a Gaussian process, ψ(⋅) is a known real-valued function, and h(⋅,⋅) is a non-negative real-valued function of the random variable that may depend on the dispersion parameter (see, e.g., Lee and Nelder, 1996). The link function g(⋅) is a key component of such models and connects the support of Y(s) to the real line. Probit and logistic link functions are customary choices for binary data.

Our intended contribution in this manuscript is to offer a scalable inferential framework for point-referenced binary spatial data, where we account for a linear fixed effect and a spatial random field. To accommodate spatial dependence while maintaining computational efficiency, we deviate from the traditional Bayesian implementations of the spatial probit model (Albert and Chib, 1993; Berrett and Calder, 2016) that uses the conditional latent variable representation (1) of the model to sample both w’s and the hyper-parameters in a Markov Chain Monte Carlo (MCMC) algorithm. We eschew Markov chain Monte Carlo (MCMC) sampling by exploiting some analytic expressions for marginal likelihood from (1) using a probit link function as proposed in Cao et al. (2022). Avoiding MCMC offers the significant added advantage of obviating some of the challenges of poor mixing of posterior sampling high-dimensional chains resulting from weak identifiability of model hyperparameters. Our approach closely aligns with the recently proposed MCMC-free approach in Cao et al. (2022). A key innovation over this work is the use of Nearest Neighbor Gaussian Process (NNGP) (Datta et al., 2016b) to approximate the conditional prediction probabilities. This reduces the computational complexity over the algorithm of Cao et al. (2022) both theoretically and empirically. We demonstrate that our approach delivers fast and exact spatial predictive inference and scales to very large data sets using a Nearest Neighbor Gaussian Process (NNGP) approximation for the covariance function corresponding to the cumulative distribution function (cdf).

The rest of this paper is organized as follows. In Section 2, we describe the method, and discuss its advantages in terms of computational overhead. In Section 3, we demonstrate the utility of the proposed approach through simulation studies and application in invasive species data. We conclude with a discussion in Section 4.

## 2 Method

Let $Y\left(s_{i}\right)$ denote the binary outcome and $X\left(s_{i}\right)$ denote the set of covariates at location i, for i=1,…,n. A probit model for the data is given by

(2) $$Y\left(s_{i}\right)=I\left(Z_{i}⩽X{\left(s_{i}\right)}^{\prime }\beta +w\left(s_{i}\right)\right)\;\text{where}\;Z_{i}\overset{iid}{∼}N(0,1)⫫\left(X\left(s_{i}\right),w\left(s_{i}\right)\right).$$

Here $w\left(s_{i}\right)$ are the spatial random effects, customarily modeled as a Gaussian Process (GP), to account for spatial correlation in the response. Modeling w(⋅)∼GP(0,C(⋅,⋅∣θ)) we have $w={\left(w\left(s_{1}\right),\ldots ,w\left(s_{n}\right)\right)}^{\prime }∼N(0,C(\theta ))$ where $C(\theta )=\left(C\left(s_{i},s_{j}|\theta \right)\right)$, and θ denotes the spatial parameters.

Letting Φ(⋅) denote the cdf of a standard normal distribution, the joint likelihood of the data is given by

(3) $$\prod_{i=1}^{n}\text{Φ}{\left(X{\left(s_{i}\right)}^{\prime }\beta +w\left(s_{i}\right)\right)}^{Y\left(s_{i}\right)}{\left(1-\text{Φ}\left(X{\left(s_{i}\right)}^{\prime }\beta +w\left(s_{i}\right)\right)\right)}^{\left(1-Y\left(s_{i}\right)\right)}\times N(w|0,C(\theta )),$$

where N(w∣0,C(θ)) denotes the density of w, given by the pdf of N(0,C(θ)). Denote $Y={\left(Y\left(s_{1}\right),\ldots ,Y\left(s_{n}\right)\right)}^{\prime }$, $X={\left(X\left(s_{1}\right),\ldots ,X\left(s_{n}\right)\right)}^{\prime }$ and ${\text{Φ}}_{n}(m,\text{Σ})$ to be the cdf of a multivariate (n−dimenional)N(0,Σ) distribution evaluated at m. As derived in Cao et al. (2022), the marginal likelihood of Y is given by

$$p(Y)=\int \prod_{i=1}^{n}\text{Φ}{\left(X{\left(s_{i}\right)}^{\prime }\beta +w\left(s_{i}\right)\right)}^{Y\left(s_{i}\right)}{\left(1-\text{Φ}\left(X{\left(s_{i}\right)}^{\prime }\beta +w\left(s_{i}\right)\right)\right)}^{\left(1-Y\left(s_{i}\right)\right)}\times N(w|0,C(\theta ))dw\;\;=\int \prod_{i=1}^{n}\text{Φ}\left(\left(2Y\left(s_{i}\right)-1\right)\left(X{\left(s_{i}\right)}^{\prime }\beta +w\left(s_{i}\right)\right)\right)\times N(w|0,C(\theta ))dw\;\;=\int {\text{Φ}}_{n}\left(DX\beta +Dw,I_{n}\right)\times N(w|0,C(\theta ))dw,\;\;\text{where}\;D=\text{diag}\left(2Y\left(s_{1}\right)-1,\ldots ,2Y\left(s_{n}\right)-1\right)\;\;={\text{Φ}}_{n}\left(DX\beta ,I_{n}+DC(\theta )D\right)\;\text{by Lemma}\;7.1\;\text{of Azzalini and Capitanio (2014)}.$$

Hence the marginal likelihood of Y is given by a multivariate normal cdf

(4) $$p(Y)={\text{Φ}}_{n}(m,\text{Σ})\;\text{where}\;m=DX\beta ,\;\text{Σ}=I_{n}+DC(\theta )D.$$

Following the separation of variables trick proposed in Genz (1992), such multivariate cdf can be evaluated as

(5) $${\text{Φ}}_{n}(m,\text{Σ})=E_{u}\prod_{i=1}^{n}u_{i}I\left(0⩽u_{i}⩽e_{i}\right)=E_{u}\prod_{i=1}^{n}e_{i}\;\text{where}\;u={\left(u_{1},\ldots ,u_{n}\right)}^{\prime }\;\text{with}\;u_{i}\overset{iid}{∼}U[0,1]$$

and the upper-limit vector $e={\left(e_{1},\ldots ,e_{n}\right)}^{\prime }$ is given by

(6) $$e_{1}=\text{Φ}\left(m_{1}/l_{11}\right),e_{i}=\text{Φ}\left(\left[m_{i}-\sum_{j=1}^{i-1}l_{ij}{\text{Φ}}^{-1}\left(u_{j}e_{j}\right)\right]/l_{ii}\right),\;\forall i⩾2.$$

Here $L=\left(l_{ij}\right)$ is the lower-triangular Cholesky factor of Σ, i.e., $\text{Σ}=LL^{\prime }$.

### 2.1 Nearest Neighbor GP Cholesky Factors

Evaluating the marginal probabilities in (4) requires computing the Cholesky factor L of $\text{Σ}=I_{n}+DC(\theta )D$ for generating the quantities in (6). Cholesky factorization typically needs $O\left(n^{3}\right)$ floating point operations or *flops*. Cao et al. (2022) reduced the complexity to $O\left(n^{5/2}\right)$ by using a tile-low-rank approximation of Σ. In this manuscript, we propose an improved $O\left(n^{2}\right)$-complexity algorithm using Cholesky factors from Nearest neighbor Gaussian Process (NNGP, Datta et al., 2016a) covariance matrices. NNGP constructs a valid joint distribution for w on a set of locations $\left\{s_{1},\ldots ,s_{n}\right\}$ by sequentially specifying $w\left(s_{i}\right)|w\left(s_{1}\right),\ldots ,w\left(s_{i-1}\right)$ only using GP spatial correlations between $w\left(s_{i}\right)$ and w(s) for m-nearest neighbors s of $s_{i}$ among $s_{1},\ldots ,s_{i-1}$. This specification was proposed as a scheme for GP likelihood approximation originally by Vecchia (1988) and was shown to correspond to a multivariate Gaussian distribution with a valid covariance matrix in Datta et al. (2016b).

The key rationale for motivation and success of NNGP as an excellent surrogate for full GP is that if the full GP covariance function monotonically decreases with distance, the m-nearest neighbors constitute the set of m locations of among $s_{1},\ldots ,s_{i-1}$ which has the highest correlation with $s_{i}$. Thus NNGP approximations are used either on the latent spatial random effects w with covariance matrix C, or, for Gaussian responses, directly on the response vector after marginalizing out w which has the covariance matrix of the form $C+\tau^{2}I,\tau^{2}$ denoting the unstructured (non-spatial) variance. If the covariance function C(⋅,⋅∣θ) monotonically decreases with distance then so do the entries of both the matrices C and $C+\tau^{2}I_{n}$ (Finley et al., 2019), and replacing either of these covariances with their respective NNGP analogs is justified and works well.

For spatial probit linear mixed model, we propose approximating the covariance matrix $\text{Σ}=I_{n}+DCD$ with a NNGP. The justification is that the off-diagonal entries of $\text{Σ}=\left(\sigma_{ij}\right)$ satisfy

$$\left|\sigma_{ij}\right|=\left|\left(2Y\left(s_{i}\right)-1\right)C\left(s_{i},s_{j}|\theta \right)\left(2Y\left(s_{j}\right)-1\right)\right|=\left|C\left(s_{i},s_{j}|\theta \right)\right|$$

as $Y\left(s_{i}\right)$’s are binary. Hence, the absolute values of the covariances of Σ still decrease with distances and the principles of using NNGP hold.

Let $\tilde{\text{Σ}}$ denotes the NNGP covariance matrix corresponding to Σ. It is well-known (Datta et al.,) that $\tilde{\text{Σ}}$ has the following properties:

$${\tilde{\text{Σ}}}^{-1}=(I-A{)}^{\prime }F^{-1}(I-A)$$

where A is a sparse strictly lower-triangular matrix and $F=\text{diag}\left(f_{1},\ldots ,f_{n}\right)$ is diagonal, both of O(n) flops. The lower-triangular Cholesky factor $\tilde{L}$ of $\tilde{\text{Σ}}$ is then given by $\tilde{L}=\left({\tilde{l}}_{ij}\right)={\left(I-A\right)}^{-1}F^{1/2}$. Letting ${\tilde{l}}_{.j}$ denote the jth column of $\tilde{L}$, and $\eta_{j}$ the n-dimensional vector with 1 at the jth position and 0’s elsewhere, one can solve for ${\tilde{l}}_{.j}$ as

(7) $${\tilde{l}}_{\cdot j}={\left(I-A\right)}^{-1}f_{j}^{\frac{1}{2}}\eta_{j}⟺(I-A){\tilde{l}}_{.j}=f_{j}^{\frac{1}{2}}\eta_{j}⟺{\tilde{l}}_{.j}=\text{trsolve}\left(I-A,f_{j}^{\frac{1}{2}}\eta_{j}\right),$$

where trsolve denotes solution of a triangular linear system. As A is strictly lower triangular with at-most O(1) entries per row, the linear system in (7) can be solved in O(n) flops (see Saha and Datta, 2018a; Datta, 2021, for the algorithm). Repeating this for j=1,…,n. the entire Cholesky factor $\tilde{L}$ can be obtained in $O\left(n^{2}\right)$ flops or in $O\left(n^{2}/K\right)$ flops if parallelized over K computing cores as the solves for ${\tilde{l}}_{.j}$ for different j’s can proceed in an embarrassingly parallel manner.

### 2.2 Predictions in Probit Model

For prediction at a new location $s_{n+1}$, let $Y^{*}={\left(Y\left(s_{1}\right),\ldots ,Y\left(s_{n}\right),Y\left(s_{n+1}\right)\right)}^{\prime }$. Define the quantities $X^{*}$, $D^{*}$ and $C^{*}$ analogous to X, D and C respectively but for the n+1 data-points. Let $m^{*}=D^{*}X^{*}\beta$ and ${\tilde{\text{Σ}}}^{*}$ denote the NNGP matrix corresponding to ${\text{Σ}}^{*}=I_{n+1}+D^{*}C^{*}D^{{*}^{\prime }}$. Then we have

(8) $$\hat{Y\left(s_{n+1}\right)}=p\left(Y\left(s_{n+1}\right)=1|Y\right)=\frac{{\text{Φ}}_{n+1}\left(m^{*},{\tilde{\text{Σ}}}^{*}\right)}{{\text{Φ}}_{n}(m,\tilde{\text{Σ}})}.$$

Evaluating the denominator using (6) requires the Cholesky factor $\tilde{L}$ of $\tilde{\text{Σ}}$ which can be computed using $O\left(n^{2}\right)$ flops via (7). For the numerator, one needs to repeat the procedure using the Cholesky factor. ${\tilde{L}}^{*}$ of ${\tilde{\text{Σ}}}^{*}$ However, another advantage of NNGP for this task is that, having computed $\tilde{L}$, one can compute ${\tilde{L}}^{*}$ in only O(n) additional flops. To see this, let $A^{*}$ and $F^{*}$ respectively denote the n+1 dimensional analogs of A and F. Then we have

(9) $$F^{*}=\text{diag}\left(F,f_{n+1}\right),A^{*}=\left(\begin{array}{cc} A & 0\\ a_{n+1}^{\prime } & 0 \end{array}\right)\;\text{and}\;{\tilde{L}}^{*}=\left(\begin{array}{cc} \tilde{L} & 0\\ a_{n+1}^{\prime }\tilde{L} & f_{n+1}^{1/2} \end{array}\right).$$

Hence, the only added computations for ${\tilde{L}}^{*}$ are computing the nearest-neighbor kriging weight-vector $a_{n+1}$ and the nearest-neighbor kriging variance $f_{n+1}$, and then computing $v^{\prime }=a_{n+1}^{\prime }\tilde{L}$. Obtaining both $a_{n+1}$ and $f_{n+1}$ are done in O(1) flops as they only involve m-dimensional vectors and matrices, m being the number of nearest neighbors. To get v, we solve the sparse lower-triangular system ${\tilde{L}}^{-1}v=a_{n+1}$ for v. This is akin to the linear system in (7) and can be done in O(n) additional flops due to sparsity of ${\tilde{L}}^{-1}=(I-A)F^{-1/2}$.

Having computed, $\tilde{L}$ and ${\tilde{L}}^{*}=\left({\tilde{l}}_{ij}^{*}\right)$ we will evaluate the ratio in (8) via (5) and (6). Let $u_{n+1}∼U[0,1]⫫]u$ and $u^{{*}^{\prime }}=\left(u^{\prime },u_{n+1}\right)$). Then, for the denominator, we first calculate the $e_{i}$’s defined in (6) by replacing $l_{ij}$ with ${\tilde{l}}_{ij}$. Subsequently, noting from (9) that $\tilde{L}$ and ${\tilde{L}}^{*}$ agree on the top n×n block, these $e_{i}$’ can also be reused for evaluating the numerator. One only needs to compute an additional

(10) $$e_{n+1}=\text{Φ}\left(\left[m_{n+1}-\sum_{j=1}^{n}{\tilde{l}}_{n+1,j}^{*}{\text{Φ}}^{-1}\left(u_{j}e_{j}\right)\right)\right]/{\tilde{l}}_{n+1,n+1}^{*}).$$

Now we can compute the prediction $\hat{Y}\left(s_{n+1}\right)$ from (8) as

(11) $$\hat{Y\left(s_{n+1}\right)}=\frac{{\text{Φ}}_{n+1}\left(m^{*},{\tilde{\text{Σ}}}^{*}\right)}{{\text{Φ}}_{n}(m,\tilde{\text{Σ}})}=\frac{E_{u^{*}}\prod_{i=1}^{n+1}e_{i}}{E_{u}\prod_{i=1}^{n}e_{i}}\approx \frac{\frac{1}{R}\sum_{r=1}^{R}\prod_{i=1}^{n+1}e_{i}^{\left(r\right)}}{\frac{1}{R}\sum_{r=1}^{R}\prod_{i=1}^{n}e_{i}^{\left(r\right)}}.$$

Here the last approximation reflects the practice where the expectation is replaced by Monte-Carlo expectation using samples $u_{i}^{\left(r\right)}$ from U[0,1] and generating the $e_{i}^{\left(r\right)}$ for r=1,…,R. The entire evaluation requires $O\left(n^{2}\right)$ flops – an improvement over the total $O\left(n^{5/2}\right)$ flops ($O\left(n^{3/2}\right)$ flops for each Monte Carlo sampling and $O\left(n^{5/2}\right)$ flops for the Cholesky factor) algorithm proposed in Cao et al. (2022).

In practice, the parameters β and θ are unknown. They can be evaluated by cross-validation as outlined in Cao et al. (2022) using the mean square error (MSE) loss. Other loss functions like the mis-classification loss or the Kullback-Leibler divergence (KLD)-loss can also be considered that accounts for the binary nature of the response.

## 3 Illustrations

In this section, we illustrate the predictive performance and the computational scalability of the probit-NNGP model proposed in Section 2.1 in large simulation experiments and real world geospatial data. The implementation makes use of code snippets from the existing R package BRISC (Saha and Datta, 2018b), and heavily leverages Intel Math Kernel Library’s threaded BLAS and LAPACK routines. In this section, we will be using 15 nearest neighbors (m in Section 2.1) and a location ordering scheme based on sum of coordinates for the NNGP based conditional probabilities derived in Section 2.

### 3.1 Simulation Experiment

For evaluating the performance of the proposed approach, we closely follow the simulation setup in Cao et al. (2022). We restrict ourselves to the scenario where β=0 and simulate data on an equispaced g×g grid on a unit square. We simulate binary response Y at these $n=g^{2}$ locations as follows:

$$Y\left(s_{i}\right)∼\text{Bernoulli}\left(\text{Φ}\left(w\left(s_{i}\right)\right)\right);\;\left(w\left(s_{1}\right),w\left(s_{2}\right),\ldots ,w\left(s_{n}\right)\right)∼N(0,C),$$

where C is the covariance matrix corresponding to exponential covariance kernel with spatial variance $\sigma^{2}$ and spatial decay ϕ, i.e.


$$C_{i,j}=\sigma^{2}\exp\left(-\phi \|s_{i}-s_{j}\|\right).$$

To demonstrate the performance of probit-NNGP in a wide spectrum of sample sizes, we use the following choices of g∈{15,25,50,100}. We first simulate the probabilities and the corresponding binary response for g=100. For all of the other choices of g, we obtain the corresponding data by subsetting the full data over the suitable equally spaced subgrid. We set the spatial parameters as follows: $\sigma^{2}=1$, $\phi =\sqrt{30}$. To assess the predictive performance of probit-NNGP, we consider two sets of 100 out-of-sample locations in Cao et al. (2022):

1. 100 random locations from the unit square
2. a grid of 100 locations, non overlapping with the training dataset.

For each choice of g, we replicate the process 100 times. Instead of using the true parameter values for prediction purposes, we first estimate the parameters and next use the estimates for prediction. We estimate the parameters by optimizing the likelihood in Section 2. Performing a global optimization with respect to the spatial parameters can lead to significant computational overhead. In Cao et al. (2022), it was demonstrated that prediction performances are robust with respect to minor variation in parameter choices. We use a grid search based optimization over $\left\{\sigma^{2},\phi \right\}$, by evaluating the likelihood (5) on a grid of values in the joint parameter space. We use a 10 × 10 grid on $\left[\sqrt{\frac{1}{2}},\sqrt{\frac{3}{2}}\right]\times [\sqrt{15},\sqrt{45}]$. We use mean squared error between the estimated predictive probabilities at the out-of-sample locations and the true ones.

In order to compare the accuracy of the proposed approach with a state-of-the-art solution for this problem, we also consider the minimax tilting method based evaluation of truncated normal (TN) distribution (Botev, 2017), implemented in R package TruncatedNormal (Botev and Belzile, 2021). The authors proposed a novel minimax tilting method to simulate i.i.d. observations from a multivariate Gaussian distribution. This provides with an efficient estimator for otherwise intractable cumulative distribution function of multivariate Gaussian distribution. Cao et al. (2022) developed a scalable probit model prediction based on Genz (1992) separation of variable algorithm. They achieve scalability through tile-low-rank (TLR) representation of the covariance matrix, to approximate the required cholesky decomposition in (6). This method is implemented with R package tlrmvnmvt (Cao et al., 2020, 2022) and the repository https://github.com/danieledurante/PredProbitGP. As far as the proposed approach (TLR) in Cao et al. (2022) is concerned, TN was used as the gold standard for accuracy whereas TLR vastly outperformed TN in computational overhead. Hence we compare the accuracy and runtime of probit-NNGP with that of both TN and TLR throughout the article. For implementing TN and TLR, we follow the model parameter choices in the Tutorial in the aforementioned repository. The packages to implement probit-NNGP, TN and TLR are implemented in R, with the base code written in C/C++/RCPP.

We set a constraint of 24 hours on the computational budget (for estimation and prediction together) and report the results for TN and TLR for values of g, where the whole prediction task for one replicate does not exceed the 24 hours. We note that both TN and TLR rely on parameter estimation by optimization of the probit likelihood. To be consistent in our empirical comparison, we use the same grid-search strategy and grid points as probit-NNGP for this estimation part.

Tables 1a and 1b demonstrate that the accuracy of the proposed approach is comparable to that of the TN for both sets of out-of-sample locations. Note that for some of the larger sample sizes, the TN method could not be completed within the total allotted time. In all the settings for which all the methods were able to be completed, the predictive performances of all the methods are nearly identical.

Next, we consider the computational overhead of the methods under consideration. The total runtimes of the concerned methods can be decomposed into two components, the first component being performing a grid search to optimize the (log)likelihood with respect to the spatial parameters. The second component consists of performing prediction on K out-of-sample locations with the parameter estimates obtained in the first step. Any NNGP based method incurs a one-time cost of determining the set of ordered nearest-neighbors for each location, which can be significantly time consuming for large datasets. Here we note that once we obtain the nearest-neighbor set, it can be used in the grid search and the subsequent prediction process. As far as prediction in out-of-sample data is concerned, unlike TN or TLR, probit-NNGP performs predictions on all locations together. Recall that, since the estimation process in probit-NNGP returns the $\tilde{L}$ in (7), the ${\tilde{L}}^{*}$ in (9) can be obtained in O(n) computation. The reported runtime for TN for prediction at an out-of-sample location in Cao et al. (2022) includes the runtime needed to evaluate the denominator in (8) (i.e. our reported runtime in Table 2a). This shows that our reported total runtime for TN is consistent with that of Cao et al. (2022) for comparable sample sizes. The TLR runtimes in Table 2b also are consistent with the reported timings in Cao et al. (2022). Here we note that unlike TN, TLR predicts both the numerator and denominator of (8) simultaneously, hence given values of spatial parameters, the total runtime for prediction at one location only involves the timing reported in Table 2b.

The runtimes reveal the superior capability of the probit-NNGP model for scaling to settings involving massive geospatial data. For each runtime, we see that the probit-NNGP is orders of magnitude faster than TN and TLR. For the largest sample size of $100^{2}=10000$, NNGP completes estimation and prediction in less than a total of 3 minutes. As we have seen from Tables 1a and 1b, this expedition in computational speed comes at no discernible compromise in terms of predictive accuracy.

In case the parameters are known, i.e. we do not need to estimate the parameters. The time to predict at K locations in this scenario are given in Table 3.

### 3.2 Invasive Species Data Analysis

We demonstrate the utility of the probit-NNGP model in analysis of binary spatial data. The dataset under consideration informs about presence or absence of an invasive plant species *Celastrus orbiculatus* in the state of Connecticut, USA. The data set consists of 603 locations; the response is a presence–absence binary indicator (0 for absence) for *Celastrus orbiculatus*. We refer the readers to Banerjee and Gelfand (2006) for details on the data. The data set contains covariates, but consistent with the theme of the present article, we focus on assessing prediction performance only using the location information and hence do not use the covariates. In order to compare the predictive performances of the concerned methods, we first scale the 603 location coordinates to [0,1]×[0,1] unit square and divide the data into 500 training and 103 out-of-sample data. For all the models, we model the dependence structure in the response variable with an exponential covariance. We first obtain a crude estimate of the spatial parameters $\left\{\sigma^{2},\phi \right\}$, by evaluating the likelihood (5) on a 25 × 10 equispaced grid on $\left[0,\sqrt{200}]\times [0,10\right]$. For TLR, the Cholesky decomposition failed on the grid search, so we only compare the performances of probit-NNGP and TN in this data. Subsequent to estimating the spatial parameters, we obtain the predictive probabilities at the 103 held-out locations corresponding to probit-NNGP and TN.

**Figure 1:**
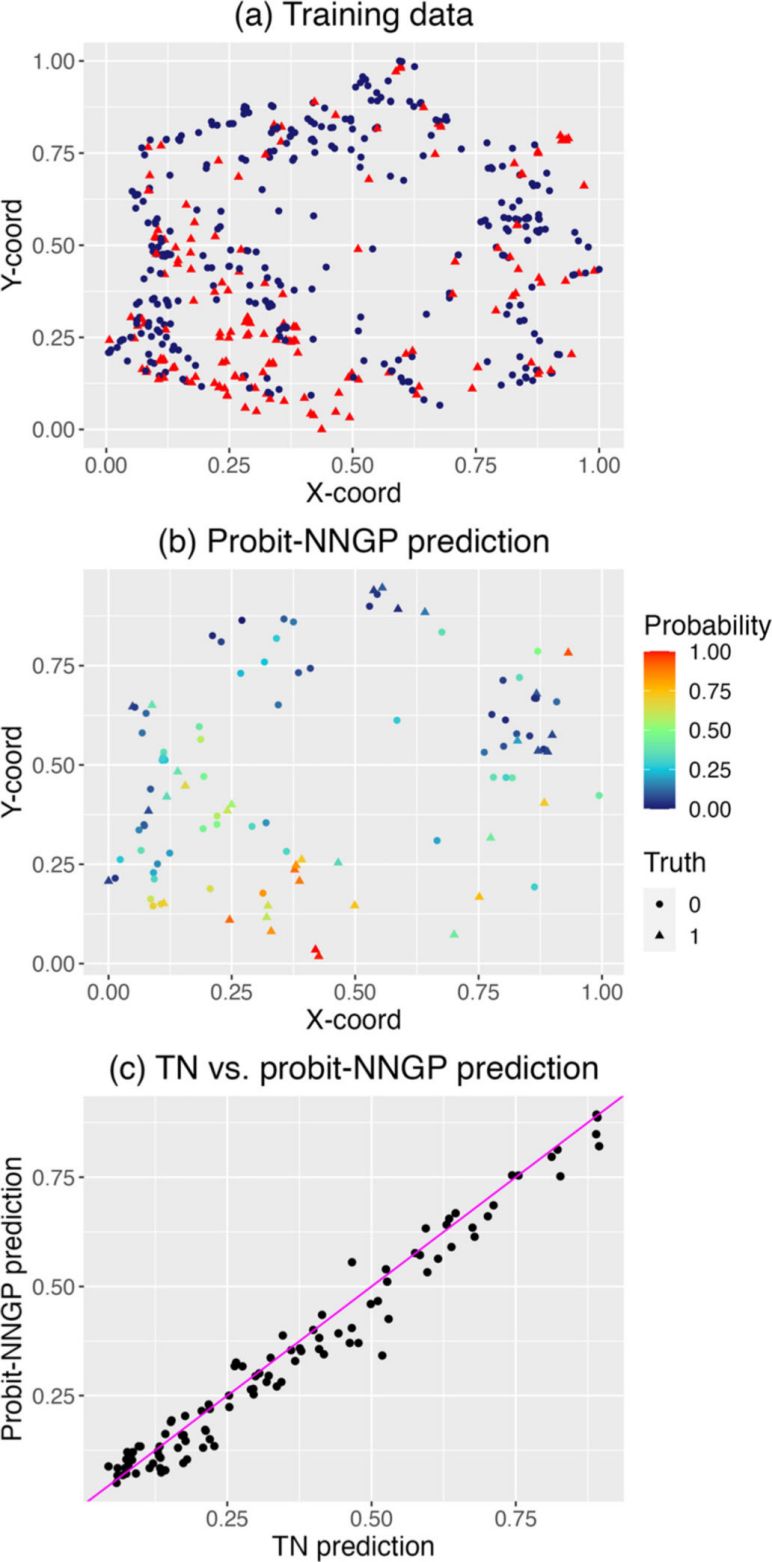
Invasive species data analysis with probit-NNGP. (a) shows the 500 training data with red denoting the presence and blue denoting the absence of *Celastrus orbiculatus*. (b) Shows the predicted probabilities of presence of *Celastrus orbiculatus*, along with the training data. (c) Shows that scatter plot of the predictions from the TN and the probit-NNGP models, demonstrating the similarity of the two.

Fig. 1 plots the training data and the predicted probabilities from probit-NNGP for the test data. The predictions are quite similar in nature with the mean square difference between the two being 0.002. This is also corroborated from the scatterplot in Panel (c) of Fig. 1 revealing the close alignment of the two sets of predictions. Unlike in the case of simulated data, we do not have access to the true probabilities at the held-out locations. Hence we measure the out-of-sample predictive performance via the area under the ROC curve (AUC) following Cao et al. (2022). Table 4 shows that the AUC for both the methods are similar. As the covariates effects for this data turned out to be statistically significant in Banerjee and Gelfand (2006), accounting for covariate effects may have lead to a better overall AUC for both the methods but would require optimization or grid-search over a higher dimensional space to obtain estimates of the regression coefficients. We also show the total runtime of probit-NNGP and TN in Table 4, which shows that probit-NNGP significantly outperforms TN in terms of computational overhead, even with the smaller sample size. Here, we note that the timing in Table 4 may seem higher than Table 2, but indeed are consistent with the ones reported in Table 2 due to the use of a denser grid in this scenario and Table 4 reporting the total runtime for all predictions as opposed to the run-time for one prediction location reported in Table 2a. For example, for TN, estimating the likelihood in (5) takes 14.1 seconds. We need to perform this operation 250 times for the grid search and 103 times for prediction at new locations. This gives us the reported time in Table 4.

## 4 Discussion

We proposed a modification of the spatial probit model prediction algorithm of Cao et al. (2022) using Nearest Neighbor Gaussian Process (NNGP) approximation. NNGP is a natural candidate for this problem as the covariance matrices involved in the probit marginal cdf’s still respect monotonicity with respect to distance – the central rational for dimension-reduction using a few nearest neighbors, and that the approach relies on Cholesky factors which are conveniently obtained for NNGP. The NNGP-based algorithm proposed here theoretically reduces the computational complexity, which is reflected in considerably improved run times in the data analysis over competing methods. We also learn that this expedition in terms of computational efficiency does not sacrifice prediction accuracy.

As suggested by one of the reviewers of the paper, we also considered using Monte Carlo (MC) simulation to estimate p(Y) directly using (4). The approach has a theoretical computational complexity of O(n), as both the approximate inverse Cholesky computation and one MC sample cost O(n). This method requires a high number of MC simulations to produce a stable estimate, as shown in the supplementary material. This makes this approach infeasible even for moderately high values of n.

One limitation of probit-NNGP is that, the cross-validation strategy only works if the number of covariates is small which limits the dimensionality of β. All the approaches (probit-NNGP, TN, TLR) perform prediction for a given set of parameters. In Cao et al. (2022) and in the present article, we use this for parameter estimation by maximising the likelihood through grid search. The overall computational time depends on the number of points in the parameters space. With zero mean, the dimension of the parameter space is equal to the number of spatial parameters, i.e. 2 for exponential covariance model. This requires a grid search over $O\left(K^{2}\right)$ points to search the parameter space, assuming K evaluation points for each parameter. If we want to account for unknown linear effect of d covariates, the dimension of the parameter space would be d+2 (d parameters denoting the effect of d covariates, i.e. β in (2) of the article). In order to get a good coverage of the parameters space, we have to perform grid search over $O\left(K^{d+2}\right)$ points, which will exponentially increase the overall cost of parameter estimation. We note that if we know the effect of the covariates, i.e. β is known or estimated apriori, the computational cost will not increase, as the unknown parameter space remains unchanged compared to the β=0 scenario. Future research will explore alternative estimation strategies for moderate or large number of covariates. Future developments may also comprise generalizing the proposed methodology for spatial multinomial probit models that can replace spatially-varying multinomial logistic regression models to predict forest type groups across large forested landscapes (Finley et al., 2009).

## Supplementary Material

Supplementary Material

## Figures and Tables

**Table 1:** MSE of predicted out-of-sample probabilities for probit-NNGP, TLR and TN, for different choices of n. Empty cells indicate the corresponding configurations to have a runtime exceeding our 24 hours computational budget.

Methods	$n=15^{2}$	$n=25^{2}$	$n=50^{2}$	$n=100^{2}$
(a) Out-of-sample random locations				
probit-NNGP	0.037	0.028	0.019	0.013
TLR	0.036	0.028	0.020	0.014
TN	0.037	0.028	–	–
(b) Out-of-sample grid locations				
probit-NNGP	0.031	0.026	0.018	0.013
TLR	0.030	0.025	0.020	0.014
TN	0.031	0.024	–	–

**Table 2:** Runtime in seconds for probit-NNGP, TLR and TN for different choices of n. Empty cells indicate the corresponding configurations to have a runtime exceeding our 24 hours computational budget.

Methods	$n=15^{2}$	$n=25^{2}$	$n=50^{2}$	$n=100^{2}$
(a) Grid search for **one** parameter combination				
probit-NNGP	0.065	0.5	9	166
TL	0.57	2.9	28	187
TN	3.5	22	–	–
(b) Prediction at **one** out-of-sample location following estimation				
probit-NNGP	< 0.01	< 0.01	< 0.01	0.025
TLR	1.2	5.8	40	271
TN	3.5	22	–	–

**Table 3:** Runtime in seconds to predict at K locations with known parameters.

Methods	$n=15^{2}$	$n=25^{2}$	$n=50^{2}$	$n=100^{2}$
probit-NNGP	0.065 + 0.01*K*	0.5 + 0.01*K*	9 + 0.01*K*	166 + 0.025*K*
TL	1.2*K*	5.8*K*	40*K*	271*K*
TN	3.5(*K* + 1)	22(*K* + 1)	-	-

**Table 4:** AUC and total (estimation + prediction) runtime in seconds for probit-NNGP and TN in *Celastrus orbiculatus* data.

Methods	AUC	Time
probit-NNGP	0.700	75
TN	0.662	4977
