# Supplementary material for: Scalable Predictions for Spatial Probit Linear Mixed Models Using Nearest Neighbor Gaussian Processes
Source: J Data Sci. Author manuscript; Available in PMC 2023 Oct 2. (PMC10544813; doi:10.6339/22-jds1073)
Supplement: Supplementary Material [file NIHMS1847490-supplement-Supplementary_Material.pdf]

Journal of Data Science xx (xx), 1–2 DOI: xx.xxxx/xxxxxxxxx
August 2022

Supplementary Materials for "Scalable Predictions for Spatial Probit Linear Mixed Models Using Nearest Neighbor Gaussian Processes"

Arkajyoti Saha[1], Abhirup Datta[2], and Sudipto Banerjee[*3]

[1]Department of Statistics, University of Washington, Seattle, WA, USA
[2]Department of Biostatistics, Johns Hopkins University, Baltimore, MD, USA
[3]UCLA Department of Biostatistics, 650 Charles E. Young Drive South, University of California Los Angeles, CA 90095-1772, USA.

Abstract

This supplementary material contains discussion on why is it infeasible to directly use a Monte Carlo sampling to estimate $p(Y)$ in (4), evaluation of the algorithms under consideration with respect to misclassification error, and details of the code and data used in the article.

1 Why is it infeasible to use a Monte Carlo sampling to directly estimate $p(Y)$ in (4)

A reviewer suggested to use a Monte Carlo sampling to estimate $p(Y)$ in (4), by first computing the sparse Cholesky approximation ($\mathcal{O}(n)$ complexity) and then performing Monte Carlo simulations from the sparse Cholesky ($\mathcal{O}(n)$ complexity). This is a very interesting suggestion, which has the potential to improve the theoretical time complexity of the procedure. The only caveat is the number of Monte Carlo simulations required to obtain a stable estimate of the truncated normal. We illustrate this with a theoretical explanation with a toy example:

Consider the case where the outcomes are independent. In our scenario, $a = -\inf$ and $b = 0$. Here, $w(s_i)$'s are generated independently. Here, using the aforementioned approach, with K MC iterations, the probability can be approximated as follows:

1. Simulate $t_1, t_2, \ldots, t_K$, with $t_i \overset{i.i.d.}{\sim} N\left(0, \tilde{\Sigma}\right)$.
2. $$p_{\mathrm{MC}} = \frac{\sum_i^K \mathbb{1}\left\{a \tilde{\leqslant} t_i \tilde{\leqslant} b\right\}}{K},$$

   where $\tilde{\leqslant}$ denotes elementwise inequality between two vectors of comparable length.
   Here, we note that in order to obtain a stable estimate, we will need $\mathcal{O}(\mathbb{E}(\Phi(z))^{-n})$ MC simulations, where z is a standard normal distribution. This follows from the fact that $\mathbb{P}(a \tilde{\leqslant} t \tilde{\leqslant} b) = \prod_j^n \Phi(t_j)$. Since t_j are i.i.d., the result follows. As the number of required simulations grows exponentially, the effective computational cost surpasses that of the proposed approach handily.
   A simulation framework: We also conduct a simulation study to see how does this pan out in case of correlated data. For any m, we set $n = m * m$ and $\sigma^2 = 1$ and $\phi = \sqrt{30}$, we simulate correlated w and use them to simulate Y. Next we use K MC iterations to estimate $p(Y)$. Here, we want to judge the stability of the estimate. Hence we do this for 100 times to obtain the standard deviation of the estimate. For each y, we obtain the ratio of

[*]Corresponding author. Email: sudipto@ucla.edu

Received August, 20222; Accepted XXXX

the standard deviation and mean of the 100 MC estimates. We do this for 100 Y and report the mean below for varied choice of K and m. As we can see, the standard deviation though well calibrated for very small n, becomes pretty significant compared to the mean, even with $100,000$ MC iterations, for $n = 4^2$. For moderately large values of n, this becomes practically infeasible due to exponential growth in required number of MC simulations to achieve similar precision.

Table 1: Ratio of Standard deviation and mean of Monte Carlo estimates while simulating directly from multivariate Normal.

Number of MC replicates	$n = 2^2$	$n = 3^2$	$n = 4^2$
1000	0.12	0.04	0.02
10000	0.75	0.24	0.14
100000	–	3.48	2.58

Here, for $n = 4^2$ and $K = 1000$, the numerator in p_{MC} becomes zero, as that event is satisfied with very low probability, hence estimate of $p(Y)$ becomes 0.

2 Comparison of misclassification rate of the proposed approaches

Table 2: Misclassification rate for $n = 15^2$ for out-of-sample random locations and grid locations

Methods	Random location	Grid location
probit-NNGP	0.32	0.32
TL	0.32	0.32
TN	0.31	0.31

3 Code availability

The code to implement probit-NNGP is available in https://github.com/ArkajyotiSaha/probit-NNGP-code. The code consists of source code and a tutorial to implement the three methods discussed in the paper alongside the data used for data analysis. The code calls functions in https://github.com/danieledurante/PredProbitGP and a "README" file is available at the link for better navigation of the files.
